# The Landscape of Cell Death Processes with Associated Immunogenic and Fibrogenic Effects in Arrhythmogenic Cardiomyopathy

**DOI:** 10.3390/jcdd9090301

**Published:** 2022-09-08

**Authors:** Wenzhao Lu, Yanfang Rao, Yao Li, Yan Dai, Keping Chen

**Affiliations:** 1 State Key Laboratory of Cardiovascular Disease, National Center for Cardiovascular Diseases, Arrhythmia Center, Fuwai Hospital, Chinese Academy of Medical Sciences & Peking Union Medical College, Beijing 100037, China; 2 School of Health, Guangzhou Huaxia Vocational College, Guangzhou 510900, China

**Keywords:** arrhythmogenic cardiomyopathy, cell death, immune response, myocardial fibrosis, RNA sequencing, bioinformatics analysis

## Abstract

Arrhythmogenic cardiomyopathy (ACM) is a heritable myocardial disease characterized by life-threatening ventricular arrhythmias and sudden cardiac death. Cardiomyocyte death is an essential pathogenic mechanism in ACM, but the cell death landscape has never been elucidated. Our study aimed to address this problem based on RNA-sequencing (RNA-seq) data. Myocardial RNA-seq data from arrhythmogenic right ventricular cardiomyopathy (ARVC) patients and normal controls were obtained from the Gene Expression Omnibus database (GSE107475, GSE107311, GSE107156, GSE107125). Signature gene sets of cell death processes, immune cells, and pathways were collected. Single-sample gene-set enrichment analysis calculated the enrichment scores for these signature gene sets. The RNA-seq data of induced pluripotent stem cell-derived cardiomyocytes (iPSC-CMs) derived from an ACM patient were used for validation (GSE115621). Weighted gene coexpression network analysis (WGCNA) was applied to identify coexpression modules. Immunogenic cell death, apoptosis, necroptosis, and pyroptosis were significantly up-regulated in ARVC. Positive correlations of these four up-regulated cell death processes with immune cells and pathways were found within the ARVC myocardium. In the ARVC sample cluster with higher cell death levels, central memory CD4 T cell, memory B cell, type 1 T helper cell, mast cell, natural killer T cell, and plasmacytoid dendritic cell were more substantially infiltrated. Similarly, immune pathways were more up-regulated in this cluster. Positive linear correlations were found between cell death, immune responses, and myocardial fibrosis within the ARVC samples. Eventually, WGCNA identified a shared coexpression module related to these mechanisms. This study first demonstrated the landscape of cell death processes in the ACM (ARVC) myocardium and their positive correlations with immune responses and myocardial fibrosis. These mechanisms have potential interactions and jointly contribute to the pathogenesis of ACM.

## 1. Background

Arrhythmogenic cardiomyopathy (ACM) is a heritable progressive myocardial disease characterized by an increased risk of malignant ventricular arrhythmias and sudden cardiac death [1]. Arrhythmogenic right ventricular cardiomyopathy (ARVC) is a common subtype of ACM, of which approximately 60% have left ventricular involvement [2]. Currently, ACM is known to be mainly caused by desmosomal gene mutations, such as plakoglobin (JUP), desmoplakin (DSP), plakophilin2 (PKP2), desmoglein2 (DSG2), and desmocollin2 (DSC2) [1]; in addition, non-desmosomal mutations have been reported to cause ACM [1]. Pathogenic mechanisms of ACM include cardiomyocyte loss, fibrofatty replacement, and immune responses or inflammation, leading to cardiac dysfunction and arrhythmogenic substrates [3]. Studies have found several mechanisms causing cardiomyocyte loss, including mechanical injury caused by desmosome dysfunction and cardiomyocyte uncoupling, sarcolemmal integrity damage, activation of programmed cell death (PCD) pathways (apoptosis or necroptosis), and immune injury [4,5]. However, the landscape of cell death processes has never been comprehensively studied. Inflammation or immune cell infiltration and fibrofatty replacement can be found around the areas where cardiomyocyte death occurs, indicating the potential associations between these procedures [6]. Immune responses can be induced by some cell death forms called immunogenic cell death (ICD), which can activate innate or adaptive immunoreaction [7]. Except for the cell death categories mentioned above, the roles of other types in ACM, such as necrosis, autophagy, pyroptosis, and ferroptosis, are unknown.

In this study, RNA-sequencing (RNA-seq) data of explanted myocardial samples from ARVC patients were used to quantify the levels of several well-defined cell death procedures while exploring the related biological processes and coexpression gene modules. We aimed to provide a novel perspective on the cell death landscape and the pathogenesis of ACM (or ARVC).

## 2. Methods

### 2.1. Collection and Processing of RNA-seq Data

The derivation and validation datasets were obtained from the Gene Expression Omnibus (GEO, https://www.ncbi.nlm.nih.gov/geo/, accessed on 16 January 2022) database. The derivation dataset contains the latest and largest RNA-seq data of right ventricular (RV) and left ventricular (LV) myocardial samples from definitively diagnosed ARVC patients undergoing heart transplantation and non-diseased donor hearts. The data were released by a research group on 20 November 2020. There are four data series: GSE107475 (nine samples of ARVC-RV), GSE107311 (six samples of ARVC-LV), GSE107156 (five samples of normal RV [N-RV]), and GSE107125 (six samples of normal LV [N-LV]). The validation dataset (GSE115621) contains the RNA-seq data of induced pluripotent stem cell-derived cardiomyocyte (iPSC-CM) samples from an ACM patient with a pathogenic homozygous mutation in PKP2 (*n* = 4) and healthy controls (*n* = 4). The sequencing platform was Illumina HiSeq 2500 (Homo sapiens). The data were transformed by robust quantile normalization and log2 (*n* + 1) before further analysis.

### 2.2. Signature Gene Sets of Cell Death, Immune Cells, and Pathways

Signature genes of seven well-defined cell death processes were gathered from the previously published literature and the Molecular Signature Database (MsigDB, https://www.gsea-msigdb.org/, accessed on 30 April 2022), including ICD (*n* = 30) [8,9], apoptosis (*n* = 87) [10,11], autophagy (*n* = 35) [10,11], necroptosis (*n* = 67) [12], necrosis (*n* = 62) [10,11], pyroptosis (*n* = 50) [13], and ferroptosis (*n* = 60) [14,15] (Supplementary data-sheet).

Signatures of 28 immune cells were obtained from previous studies [16,17], including activated B cell, activated CD4 T cell, activated CD8 T cell, central memory CD4 T cell (CD4 T_CM_), central memory CD8 T cell, effector memory CD4 T cell, effector memory CD8 T cell, gamma delta T cell, immature B cell, memory B cell (MBC), regulatory T cell (Treg), T follicular helper cell, type 1 T helper cell (Th1), type 17 T helper cell (Th17), type 2 T helper cell (Th2), activated dendritic cell (ADC), CD56bright natural killer (NK) cell, CD56dim NK cell, eosinophil, immature DC (IDC), macrophage, mast cell, myeloid-derived suppressor cell (MDSC), monocyte, NK cell, NKT cell, neutrophil, and plasmacytoid DC (PDC) (Supplementary data-sheet). In addition, gene sets representing 17 immune pathways were collected from the Kyoto Encyclopedia of Genes and Genomes (KEGG) database (https://www.genome.jp/kegg/, accessed on 29 April 2022) (Supplementary data-sheet).

### 2.3. Differentially Expressed Cell Death Signature Genes

Differentially expressed genes (DEGs) between the ARVC and normal myocardial samples were screened using the limma (3.50.3) package in R (4.1.3). DEGs were defined as genes with a false discovery rate (FDR) <0.05. The DEGs were overlapped with the seven signature gene sets of cell death to identify the differentially expressed cell death signature genes (CD-DEGs). Protein–protein interaction (PPI) network of the CD-DEGs was generated in the STRING database (https://string-db.org/, accessed on 8 May 2022) and visualized by the Cytoscape (3.7.2) software. The nodes’ connection degrees within the PPI network were calculated by CytoHubba (0.1) to evaluate the hub genes.

### 2.4. Comprehensive Analysis of Cell Death and Immune Responses Based on ssGSEA

The ssGSEA is an algorithm used to calculate the enrichment score (ES) of a given gene set in a sample based on gene expression levels; the ES reflects the relative abundance or activation status of the gene set [18]. Such a gene set can be the components or molecular markers of a biological process or a cell type. We calculated the cell death ESs for each sample in the derivation dataset with inter-sample standardization. Then, the standardized cell death ESs were compared between the ARVC and normal groups to identify the up-regulated cell death forms in ARVC. Pearson’s correlation coefficients (PCCs) and linear regression models were computed between each two cell death processes within the ARVC samples. A *p*-value < 0.05 was considered significant. Similarly, ssGSEA was repeated in the validation dataset to confirm whether similar differences in cell death exist between the iPSC-CM samples from the ACM patient and healthy controls.

On the other hand, the standardized ESs of immune cells and pathways were also calculated to represent the levels of immune cell infiltration and pathway activity. Inter-group comparisons were performed to identify the highly infiltrated immune cells and up-regulated pathways in ARVC. Within the ARVC samples, correlation analysis was performed between the up-regulated cell death processes and the discrepant immune cells and pathways.

Furthermore, the ARVC samples were divided into two clusters by principal component analysis (PCA) and K-means clustering based on the standardized ESs of the up-regulated cell death processes in ARVC. Levels of cell death, immune cell infiltration, and immune pathways were compared between the two clusters. Besides, differential expression analysis was also conducted. Functional enrichment analysis for the DEGs, including the Gene Ontology biological processes (GO-BP) and KEGG pathways, was completed by Metascpae (https://metascape.org/, accessed on 7 May 2022).

### 2.5. Identification of Functional Coexpression Modules by WGCNA

Weighted gene coexpression network analysis (WGCNA) is an integrated algorithm to identify clusters of tightly connected genes based on their expression levels [19]. DEGs between the ARVC and normal groups were used for WGCNA. The network type was set to “signed hybrid”, while the Pearson’s correlation was applied to construct the network. An R-square of 0.8 was set to select the soft threshold. The minimum module size and dendrogram cutting threshold were set to 30 and 0.2 for module calculation and combination. Module eigengenes were used to represent the modular expression levels. Intra-modular hub genes were defined as module membership (MM) and gene significance (GS) >0.6.

### 2.6. Additional Methods and Statistics

Inter-group comparisons of enrichment scores were evaluated by independent-sample t test, with Welch correction for unequal variances and a significance level of 0.05. Two centers were set for K-means clustering with the Hartigan–Wong algorithm by default. Markov clustering (MCL) was used to identify clusters in PPI networks. Transcription regulators targeting given genes were analyzed in the TRUST database (https://www.grnpedia.org/trrust/, accessed on 18 June 2022). Data analysis and visualization were completed in R (version 4.1.2) and RStudio (2021.09.2 + 382), with the limma (3.50.3), GSVA (1.42.0), preprocessCore (1.56.0), pheatmap (1.0.12), and ggplot2 (3.3.5) packages.

## 3. Results

### 3.1. Differentially Expressed Cell Death Signature Genes

**Figure 1:**
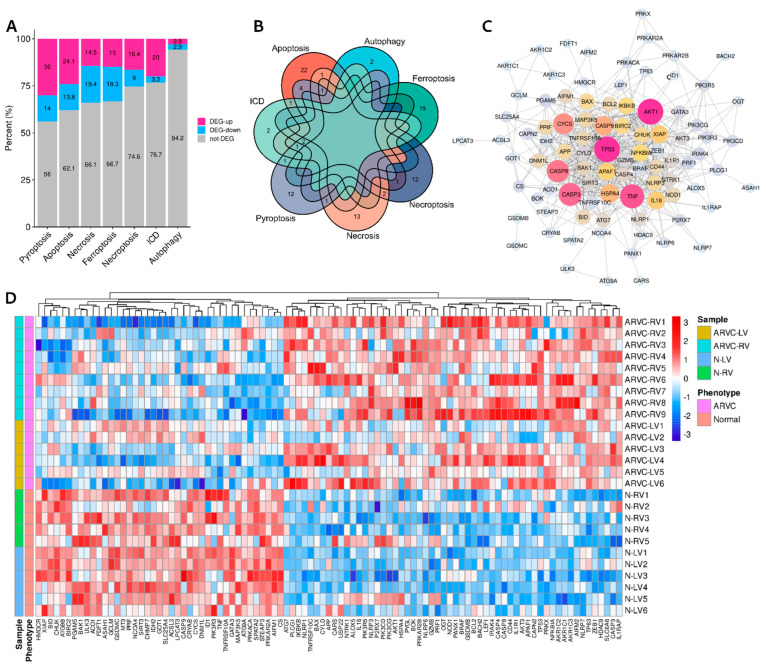
Differentially expressed cell death signature genes (CD-DEGs). (**A**): Proportions of the up-regulated and down-regulated CD-DEGs. (**B**): Overlaps of the CD-DEGs. (**C**): Protein–protein interaction network of the CD-DEGs. (**D**): A heatmap shows the CD-DEGs between ARVC samples and normal controls. ARVC, arrhythmogenic right ventricular cardiomyopathy; ARVC-LV/RV, left/right ventricular myocardial samples from ARVC patients; N-LV/RV, left/right ventricular myocardial samples from normal controls; ICD, immunogenic cell death.

Differential expression analysis identified 5375 DEGs (ARVC vs. normal), among which 2530 genes were up-regulated while 2845 were down-regulated in the ARVC myocardium. After overlapping with the cell death signature genes, different proportions of DEGs were recognized in each cell death category (Table 1, Figure 1A). The proportions of up-regulated DEGs in pyroptosis (30% vs. 14%), apoptosis (24.1% vs. 13.8%), necroptosis (16.4% vs. 9%), and ICD (20% vs. 3.3%) were higher than down-regulated ones; however, the proportions of up-regulated DEGs in necrosis (14.5% vs. 19.4%), ferroptosis (15% vs. 18.3%), and autophagy (2.9% vs. 2.9%) were lower or equal in contrast to those down-regulated. ICD, apoptosis, necroptosis, pyroptosis, and necrosis had overlapped DEGs with each other (Figure 1B, Supplementary Table S1). The ARVC samples had more up-regulated CD-DEGs than the normal controls (Figure 1D). The PPI network of CD-DEGs is illustrated in Figure 1C, in which top-10 genes ranked by node degrees were AKT1, TP53, TNF, CASP3, CASP8, CYCS, CASP9, HSPA4, IL18, and XIAP.

### 3.2. Comparisons and Correlation Analysis of Cell Death Processes

**Figure 2:**
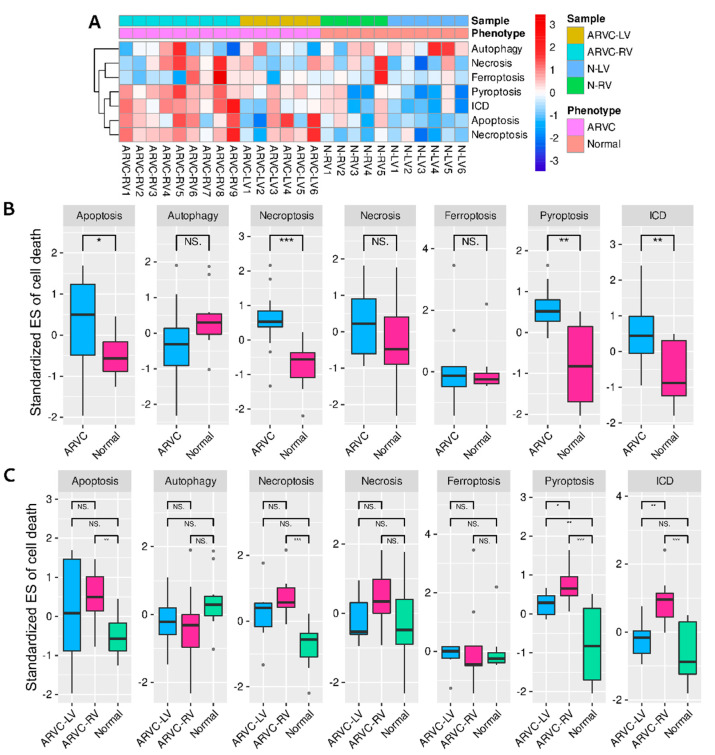
Single-sample gene-set enrichment analysis (ssGSEA) of cell death processes. (**A**): A heatmap shows the standardized enrichment scores (ES) of each sample. (**B**): Comparisons of the standardized cell death ESs between the ARVC and normal groups. (**C**): Comparisons of the standardized cell death Ess, among ARVC-LV, ARVC-RV, and normal controls. Abbreviations are the same as in Figure 1. *** *p* < 0.001; ** *p* < 0.01; * *p* < 0.05; NS, non-significance.

The standardized ESs of seven cell death processes for each sample are shown in Figure 2A. The ARVC myocardial samples exhibited generally higher cell death levels than the normal controls. Inter-group comparisons indicated that apoptosis, necroptosis, pyroptosis, and ICD were significantly up-regulated in ARVC (Figure 2B). We further compared the cell death levels between the ARVC-RV, ARVC-LV, and normal samples considering potential LV involvement and found significantly higher levels of apoptosis, necroptosis, and ICD in ARVC-RV than in normal controls, with similar trends in ARVC-LV; only pyroptosis exhibited significantly higher levels in both ARVC-RV and ARVC-LV than in the control group (Figure 2C). The results indicated that both ARVC-RV and ARVC-LV had analogical cell death profiles, and the general degrees of ARVC-LV were lower than ARVC-RV but higher than normal controls.

Similar results were generated from the validation dataset. The iPSC-CM samples of ACM showed higher levels of apoptosis, necroptosis, ICD, and ferroptosis than the heath controls, but there was no apparent difference in pyroptosis (Supplementary Figure S1). Since iPSC-CM data eliminated the influence of non-myocardial cells, it was reasonable to consider these cell death processes to occur in cardiomyocytes.

Subsequently, we found positive correlations between ICD and apoptosis (PCC = 0.55, β = 0.43, *p* = 0.03), ICD and necroptosis (PCC = 0.74, β = 0.78, *p* = 0.002), and apoptosis and necroptosis (PCC = 0.83, β = 1.11, *p* = 0.0001) within the ARVC myocardium, whereas the correlation between ICD and pyroptosis was marginally significant (PCC = 0.49, β = 0.86, *p* = 0.06) (Supplementary Figure S2).

### 3.3. Relationships between Cell Death and Immune Responses

**Figure 3:**
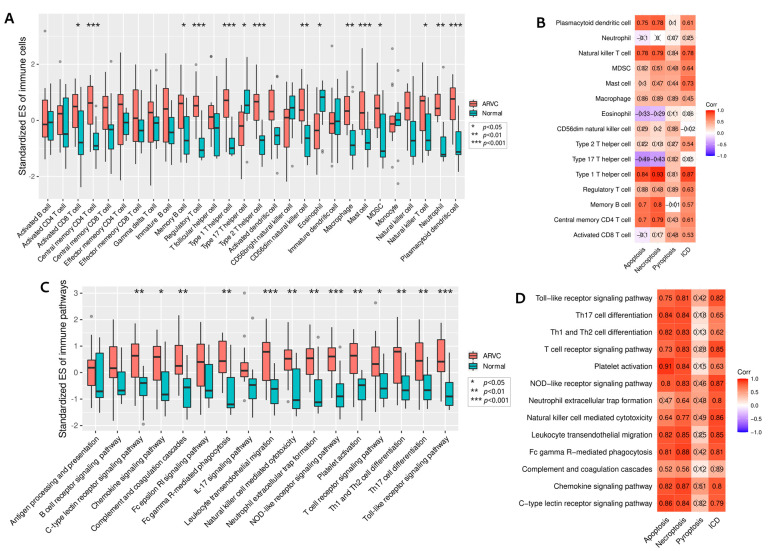
SsGSEA of immune cells and pathways. (**A**): Comparisons of the standardized ESs of 28 immune cells between two groups to identify the highly infiltrated immune cells in ARVC. (**B**): Correlations between the levels of cell death and immune cells. (**C**): Comparisons of the standardized ESs of 17 immune pathways between two groups to identify the up-regulated pathways in ARVC. (**D**): Correlations between the levels of cell death and immune pathways. MDSC, myeloid-derived suppressor cell; Th, T helper cell. Other abbreviations are the same as in Figure 1 and Figure 2. The symbol “×” means insignificant correlation.

The standardized ESs of 28 immune cell types were calculated. The overall infiltration levels of ARVC samples were higher than normal controls (Supplementary Figure S3A). Inter-group comparisons identified 13 highly infiltrated immune cells in the ARVC myocardium, including activated CD8 T cell, CD4 T_CM_, MBC, Treg, Th1, Th2, CD56dim NK cell, macrophage, mast cell, MDSC, NKT cell, neutrophil, and PDC; only Th17 and eosinophil were depressed in ARVC (Figure 3A). Figure 3B shows the correlations between the up-regulated cell death processes and the differentially infiltrated immune cells within the ARVC samples. ICD was significantly positively correlated with activated CD8 T cell, CD4 T_CM_, MBC, Treg, Th1, Th2, mast cell, MDSC, NKT cell, and PDC. Apoptosis and Necroptosis were positively correlated with CD4 T_CM_, MBC, Th1, NKT cell, and PDC. Only pyroptosis demonstrated no significant correlations with the immune cells, but its correlations with activated CD8 T cell, CD4 T_CM_, mast cell, and MDSC were relatively higher (PCC > 0.4) than with other immune cells.

On the other hand, 13 immune pathways were found significantly up-regulated in the ARVC group (Figure 3C, Supplementary Figure S3B). These pathways included c-type lectin receptor signaling pathway, chemokine signaling pathway, complement and coagulation cascades, Fc gamma R-mediated phagocytosis, leukocyte transendothelial migration, natural killer cell-mediated cytotoxicity, neutrophil extracellular trap formation, NOD-like receptor signaling pathway, platelet activation, T-cell receptor (TCR) signaling pathway, Th1 and Th2 cell differentiation, and Toll-like receptor signaling pathway. General positive correlations between these up-regulated immune pathways and the up-regulated cell death processes were found within the ARVC myocardial samples (Figure 3D).

### 3.4. Clustering of ARVC Samples Based on Cell Death Scores

**Figure 4:**
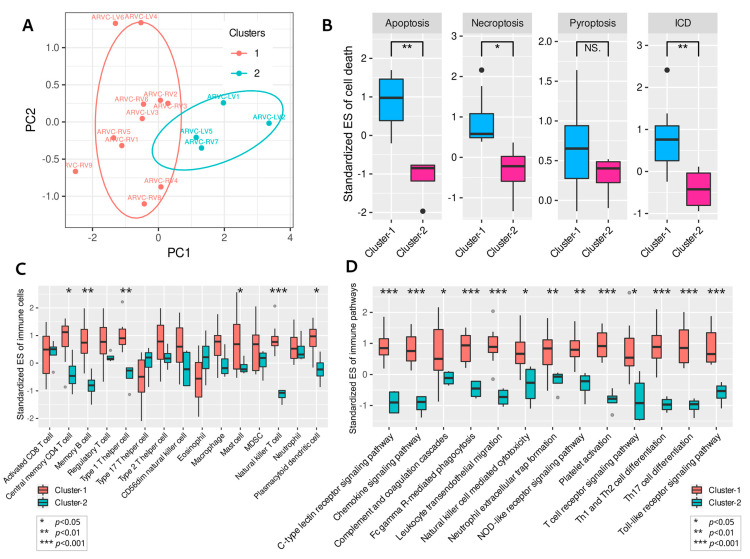
Clustering of ARVC samples based on the up-regulated cell death processes. (**A**): Principal component analysis and K-means clustering of the ARVC samples. (**B**): Comparisons of the four up-regulated cell death processes in ARVC between cluster-1 and cluster-2. (**C**): Comparisons of the highly infiltrated immune cells in ARVC between cluster-1 and cluster-2. (**D**): Comparisons of the up-regulated immune pathways in ARVC between cluster-1 and cluster-2. Abbreviations are the same as in Figure 1, Figure 2 and Figure 3. *** *p* < 0.001; ** *p* < 0.01; * *p* < 0.05; NS, non-significance.

According to the standardized ESs of ICD, apoptosis, necroptosis, and pyroptosis, PCA and K-means clustering classified the ARVC samples into two clusters (cluster-1 and cluster-2) (Figure 4A). Cluster-1 included 11 samples (ARVC-RV1, ARVC-RV2, ARVC-RV3, ARVC-RV4, ARVC-RV5, ARVC-RV6, ARVC-RV8, ARVC-RV9, ARVC-LV3, ARVC-LV4, ARVC-LV6), while cluster-2 contained four samples (ARVC-RV7, ARVC-LV1, ARVC-LV2, ARVC-LV5). Comparing the four cell death processes between the two clusters, we found that cluster-1 had significantly higher levels of apoptosis, necroptosis, and ICD than cluster-2, with a similar trend in pyroptosis (Figure 4B). Consequently, cluster-1 was considered to contain samples with higher cell death intensity.

The highly infiltrated immune cells in ARVC were also compared. The infiltration levels of CD4 T_CM_, MBC, Th1, mast cell, NKT cell, and PDC in cluster-1 were significantly higher than in cluster-2 (Figure 4C). Others, such as Treg, Th2, CD56dim NK cell, macrophage, and MDSC, also tended to be more highly infiltrated in cluster-1 (Figure 4C). Accordingly, the levels of 13 immune pathways were significantly up-regulated in cluster-1 (Figure 4D).

**Figure 5:**
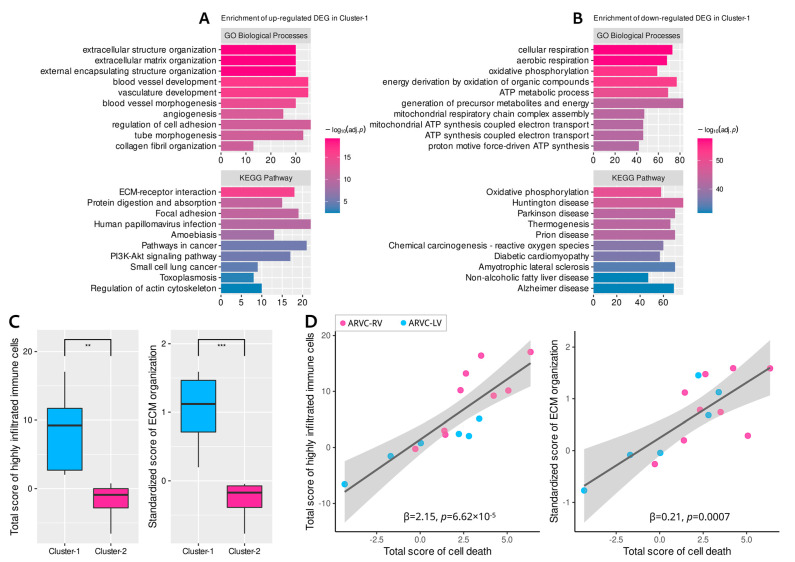
Enrichment analysis of the differentially expressed genes (DEGs) between the two clusters and functional correlation analysis. (**A**): Enrichment results of the up-regulated DEGs in cluster-1. (**B**): Enrichment results of the down-regulated DEGs in cluster-1. (**C**): Comparisons of the total scores of the highly infiltrated immune cells in ARVC and fibrosis activity (ECM organization) between the two clusters. (**D**): Positive linear correlations of cell death levels with immune cell infiltration and fibrosis. ECM, extracellular matrix. Other abbreviations are the same as in Figure 1 and Figure 2. *** *p* < 0.001; ** *p* < 0.01.

Differential expression analysis between the two clusters identified 279 up-regulated DEGs and 877 down-regulated DEGs in cluster-1. Functional enrichment analysis indicated that the up-regulated DEGs in cluster-1 were associated with extracellular matrix (ECM) organization, collagen fibril synthesis, angiogenesis, and cell adhesion (Figure 5A), while the down-regulated DEGs were enriched in cellular respiration, energy metabolism, ATP synthesis, and oxidative phosphorylation (biological processes related to cellular survival) (Figure 5B).

### 3.5. Correlations between Cell Death, Immune Responses, and Fibrosis in ARVC

Since the biological processes related to ECM organization or collagen fibril synthesis were significantly enriched in the up-regulated DEGs of cluster-1, it was reasonable to consider that the ARVC samples in cluster-1 possessed higher myocardial fibrosis activity. Standardized ESs for the GO-BP item “ECM organization” were computed to represent the fibrotic activity of each sample. The ICD, apoptosis, necroptosis, and pyroptosis scores were added up to quantify the general level of cardiomyocyte death for each sample; analogically, the total score of the highly infiltrated immune cells in ARVC was also calculated to represent the overall intensity of immune responses.

Comparisons showed significantly higher fibrosis (*p* = 0.0004) and immune cell infiltration (*p* = 0.002) levels in cluster-1 (Figure 5C). Then we found positive linear correlations of the overall cell death levels with the total intensity of immune responses and fibrosis within the ARVC myocardium (Figure 5D). These results indicated the close linkages and potential interactions or modulations among these mechanisms in ACM.

### 3.6. Identifying the Functional Coexpression Module

**Figure 6:**
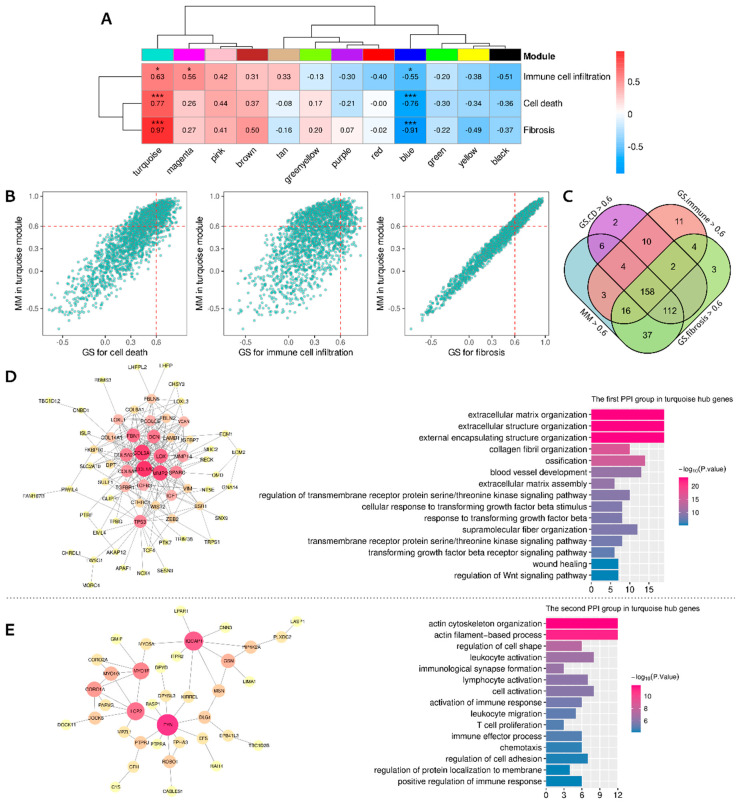
Weighted gene coexpression network analysis identified the shared correlated module. (**A**): Pearson’s correlations between the modules and the total scores of cell death, immune cell infiltration, and fibrosis in the ARVC myocardial samples. (**B**): Identifying hub genes in the turquoise module by gene significance and module membership. (**C**): The overlaps of candidate hub genes; 158 hub genes were finally identified. (**D**): The first PPI group of the hub genes with functional enrichment. (**E**): The second PPI group of the hub genes with functional enrichment. *** *p* < 0.001; * *p* < 0.05.

**Figure 7:**
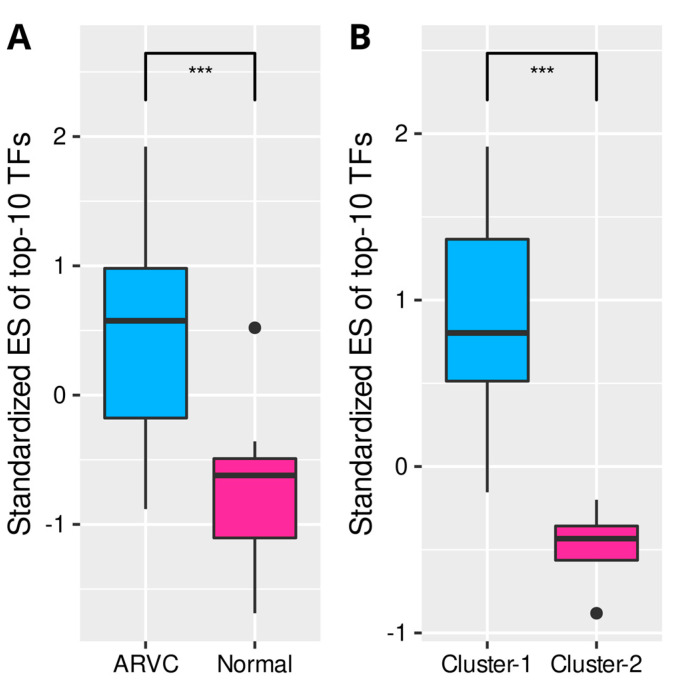
Comparisons of the standardized ESs of top 10 transcription regulators (TFs) targeting the turquoise-module hub genes. (**A**): The TFs were significantly up-regulated in the ARVC myocardial samples. (**B**): The TFs were significantly up-regulated in cluster-1 containing ARVC myocardial samples with higher cell death levels. ES, enrichment score. *** *p* < 0.001.

The 5375 DEGs between the ARVC and normal groups were put into WGCNA to construct a scale-free (R^2^ = 0.79) coexpression network with the optimized soft threshold of 10; twelve modules were eventually identified (Supplementary Figure S4, Supplementary data-sheet). Pearson’s correlation analysis identified a module (turquoise) most positively correlated with the total scores of cell death, immune cells, and fibrosis, containing 1993 genes (Figure 6A). According to the definition of intramodular hub genes, 158 candidates were identified (Figure 6B,C). The PPI network of these hub genes was obtained from the STRING database and clustered by the MCL algorithm, recognizing two primary groups containing 65 and 38 genes, respectively (Supplementary Figure S5). PPI networks of the two groups with corresponding biological functions are displayed in Figure 6D,E. The top five genes in the first PPI group ranked by node degrees were COL1A2, COL3A1, MMP2, LOX, and FBN1, while they were FYN, IQGAP1, LCP2, MYO1F, and CORO1A in the second group. The first group was enriched in the functions of extracellular matrix organization, collagen fibril organization, wound healing, serine/kinase signaling pathway, transforming growth factor-beta (TGF-β) signaling, and Wnt signaling. In contrast, the second group was enriched in immunological processes, such as leukocyte or lymphocyte activation and migration, immunological synapse formation, T-cell proliferation, chemotaxis, adhesion, and positive regulation of immune responses. Finally, transcription regulators (TFs) targeting the hub genes were searched in the TRUST database (Supplementary data-sheet). The top 10 TFs ranked by FDR were HIF1A, TFAP2C, TP53, VHL, ETV4, STAT3, HDAC2, LEF1, and NCOR1 (Table 2). The ssGSEA confirmed that these top 10 TFs were significantly up-regulated in the ARVC myocardial samples and cluster-1 (Figure 7).

## 4. Discussion

This study first demonstrated the landscape of the potentially existing cell death processes of cardiomyocytes in ACM (ARVC) and their relationships with immune responses and myocardial fibrosis based on RNA-seq data and integrated bioinformatics analysis. Cell death is an essential biological process during organism growth, development, and diseases. There are several well-defined categories of cell death, such as apoptosis, necroptosis, necrosis, autophagy, pyroptosis, ferroptosis, and ICD [7]. Previous studies have suggested that cardiomyocyte loss or death is a vital step in the pathogenesis of ACM, but limited cell death forms have been explored [1]. Several studies have demonstrated the existence of apoptosis in patients’ myocardial samples that contributed to cardiomyocyte death [20,21,22]. Research in iPSC-CM derived from ACM patients also found cell apoptosis due to desmosome dysfunction [23]. In addition, the histological features of necrosis with inflammatory infiltration were found in mouse models that overexpressed a dominant-negative Dsg2 transgene [24] and Jup mutants [25], but whether a programmed necroptosis procedure exists remains unknown. However, other PCD forms, such as autophagy, pyroptosis, ferroptosis, and ICD, have never been reported.

In this study, we collected signature gene sets of seven cell death processes and explored their activation status in ARVC and normal myocardial samples. The ARVC samples owned higher expression levels of cell death signature genes than the normal controls, especially those of apoptosis, necroptosis, pyroptosis, and ICD. Inter-group comparisons confirmed that these four cell death processes were dominantly up-regulated in the ARVC myocardium. Validation based on the RNA-seq data from iPSC-CM produced similar results, further suggesting the dominant role of apoptosis, necroptosis, and ICD in cardiomyocyte death in ACM because iPSC-CM was obtained from an ACM patient and induced to express cardiomyocyte phenotype that could reflect the pathogenic changes of the cardiomyocyte in ACM myocardium. Pyroptosis showed no difference between the two iPSC-CM groups possibly due to the limited sample size or its low activity, which requires further exploration. In the ARVC myocardium, apoptosis, necroptosis, and ICD were significantly and positively correlated. The correlation between ICD and pyroptosis was marginally due to an outlier. Apoptosis, necroptosis, and pyroptosis are essential PCD forms with cross talk and shared signal transduction molecules [26]. Apoptosis is a non-lytic cell death form with the effector molecules, such as caspase-8, caspase-9, caspase-3, and caspase-7 [7]. Necroptosis is a lytic form of cell death, as it occurs under the assembly of an activated MLKL complex that disrupts the plasma membrane and leads to cell lysis [7]. Pyroptosis is also the lytic form of cell death that creates a large pore on the cell membrane by gasdermin D (GSDM-D) or GSDM-E [7]. A previous review summarized that apoptosis interacts with necroptosis based on the activity of caspase-8 while communicating with pyroptosis by caspase-1, caspase-8, caspase-3, and GSDM-E [26]. Moreover, pyroptosis can be initiated by the NLRP3 inflammasome, which is activated in response to changes of cellular ion homeostasis secondary to MLKL-mediated membrane disruption [26].

Immune cell infiltration has been discovered around the necrotic and fibrotic regions in the ACM myocardium, such as CD45+ lymphocytes, T cells, neutrophils, macrophages, and mast cells [6]. We found 13 highly infiltrated immune cells (which have been elucidated in our previous study) [27] and 13 up-regulated immune pathways in the ARVC myocardial samples. Subtypes of T cells were the dominant infiltration component. The up-regulated immune pathways in ARVC correspond to the functions of the highly infiltrated immune cells, such as T-cell differentiation, TCR signaling, neutrophil extracellular trap formation, natural killer cell-mediated cytotoxicity, leukocyte migration, chemotaxis, and phagocytosis. Notably, ARVC samples exhibited a lower infiltration level of Th17 than normal controls, while the IL-17 signaling pathway and Th17 differentiation were up-regulated in ARVC. One possible reason for this contradictory phenomenon is the plasticity of Th17 to other subtypes of T cells [28], but the mechanisms and roles in ACM remain to be explored. Subsequently, we discovered positive correlations between cell death procedures and immune cells or pathways in the ARVC samples, especially ICD. ICD refers to some PCD forms that are adequate to activate innate or adaptive immune responses against endogenous or exogenous antigens exposed by dying cells. These endogenous antigens commonly refer to damage-associated molecular patterns (DAMPs) that are recognized by immune cells to initiate immunoreaction and establish immunological memory [7,29].

These results indicated the linkage between cell death and immune responses in ACM hearts, which was elucidated by further analysis. Sample clustering identified two clusters with discrepant cell death levels. The cluster with higher cell death intensity simultaneously possessed more elevated immune cell infiltration levels and immune pathway activity. One rational explanation is that necroptosis and pyroptosis, even apoptosis in some contexts, can release DAMPs and trigger immune responses or inflammation, exhibiting the immunogenic effect [26]. Moreover, up-regulated fibrosis activity was discovered in this cluster, and a positive linear correlation was found between cell death and fibrosis. Fibrosis is an ordinary repair method against cardiomyocyte injury and death in cardiovascular diseases [30,31,32]; thus, it is rational to consider myocardial fibrosis as the concomitant process of cardiomyocyte death in ACM. Besides, Cluster-1 was associated with pathways about cell adherence, which is essential for immune cell migration and immunoreaction.

Finally, we identified a shared coexpression module positively correlated with cell death, immunoreaction, and myocardial fibrosis within the ARVC myocardium. Intra-modular hub genes were separated into two PPI groups related to fibrosis and immune responses. Moreover, the first PPI group was associated with the TGF-β and Wnt signaling pathways that contribute to fatty replacement and myocardial fibrosis in ACM [1]. The top five genes in the first PPI group express components of collagen fibrils or ECM (COL1A2, COL3A1, FBN1) [33,34] and enzymes required for collagen synthesis, organization, and remodeling (MMP2, LOX) [35,36]. The top two genes in the second PPI group were FYN and LCP2. FYN encodes a member of the protein-tyrosine kinase family that plays a role in immunoreaction, such as T-cell differentiation and proliferation initiated by TCR stimulation [37]. LCP2 encodes an adapter protein participating in the TCR-activated protein-tyrosine kinase pathway [38]. This result further indicated the close associations and potential interactions between these mechanisms. Cardiomyocyte death is the primary pathological change in ACM, triggering immune responses or inflammation that deteriorate myocardial injury. Myocardial fibrosis plays a role in tissue repair secondary to cardiomyocyte death and immune injury. Eventually, transcription regulators targeting the intra-modular hub genes were predicted and confirmed to be up-regulated in ARVC samples and the cluster with higher cell death levels, corresponding to the correlation between the module and cell death.

As a rare disease, ACM myocardial tissue can only be obtained during heart transplantation or postmortem examination, leading to the data and clinical information limitations in the GEO database. However, it was definite that the diseased myocardial samples were from end-stage ARVC patients undergoing heart transplantation. Thus the results mainly reflected the pathogenesis at the advanced stage of ACM, which requires future experimental validation.

## 5. Conclusions

This bioinformatics study first portrayed the landscape of cell death processes in the ACM (ARVC) myocardium and their relationships with immune responses and myocardial fibrosis. The primary cell death forms in ACM include ICD, apoptosis, necroptosis, and pyroptosis. Higher cell death levels relate to a greater extent of immune responses and fibrosis. These mechanisms together shape the pathological features of ACM and may be considered the potential intervention targets.

## Figures and Tables

**Table 1:** Differentially expressed cell death signature genes.

Cell Death Category	Up-Regulated in ARVC Samples	Down-Regulated in ARVC Samples
Immunogenic cell death (*n* = 30)	PRF1, IL1R1, P2RX7, CASP8, NLRP3, BAX (*n* = 6)	TNF (*n* = 1)
Apoptosis (*n* = 87)	TNFRSF10C, NTRK1, NFKBIA, IL1R1, PIK3R5, PRKAR2B, BCL2, IL1RAP, CASP8, IKBKB, PIK3CG, AKT3, PIK3CD, CASP3, BAX, APAF1, IRAK4, TP53, AKT1, PRKX, CAPN2 (*n* = 21)	TNF, PRKACA, BID, AIFM1, CYCS, PIK3R3, TNFRSF10A, CASP9, PRKAR2A, BIRC2, CHUK, XIAP (*n* = 12)
Necroptosis (*n* = 67)	LEF1, HDAC9, PANX1, BACH2, BCL2, CASP8, CYLD, BRAF, HSPA4, USP22, APP (*n* = 11)	TNF, IDH2, SPATA2, GATA3, ID1, SIRT3 (*n* = 6)
Pyroptosis (*n* = 50)	NLRP6, NLRP7, GZMB, TP63, NLRP1, GSDMB, IL18, CASP8, NLRP3, CASP3, BAX, CASP4, NOD1, PLCG1, TP53 (*n* = 15)	GSDMC, TNF, PRKACA, CYCS, CASP9, BAK1, CHMP7 (*n* = 7)
Necrosis (*n* = 62)	NLRP6, SLC6A6, BOK, PYGL, CASP8, BAX, TP53, CYLD, OGT (*n* = 9)	ATG9B, MT3, TNF, SPATA2, PPIF, SLC25A4, MAP3K5, PGAM5, BIRC2, ATG9A, ASAH1, DNM1L (*n* = 12)
Ferroptosis (*n* = 60)	AKR1C2, AKR1C1, CD44, AKR1C3, ALOX5, ZEB1, AIFM2, TP53, CARS (*n* = 9)	LPCAT3, CS, STEAP3, GOT1, CRYAB, NCOA4, GCLM, ACSL3, ACO1, FDFT1, HMGCR (*n* = 11)
Autophagy (*n* = 35)	ATG7 (*n* = 1)	ULK3 (*n* = 1)

ARVC, arrhythmogenic right ventricular cardiomyopathy.

**Table 2:** Key transcription regulators (TF) targeting the hub genes in turquoise module.

Key TF	Description	*p*-Value	FDR	List of Overlapped Genes
HIF1A	Hypoxia inducible factor 1, alpha subunit	0.000005	0.0002	MMP2, RECK, LOX, VIM, TGFB3, NT5E, TWIST2
TFAP2C	Transcription factor AP-2 gamma	0.00006	0.001	MMP2, ESR1, ECM1
TP53	Tumor protein p53	0.0004	0.006	TP53, CABLES1, VCAN, TRIM22, ESR1, APAF1, MMP2
VHL	Von Hippel-Lindau tumor suppressor, E3 ubiquitin protein ligase	0.0005	0.006	SPARC, TCF4, TP53
ETV4	Ets variant 4	0.0007	0.006	MMP14, VIM, MMP2
STAT3	Signal transducer and activator of transcription 3	0.001	0.007	MMP2, NOX4, AKAP12, TWIST2, TP53, MMP14
HDAC2	Histone deacetylase 2	0.001	0.007	COL1A2, IGF1, APAF1
LEF1	Lymphoid enhancer-binding factor 1	0.002	0.007	ESR1, TCF4, NT5E
NCOR1	Nuclear receptor corepressor 1	0.001	0.007	IGF1, ESR1

FDR, false discovery rate.

## Data Availability

The RNA-seq data can be downloaded from the GEO database (https://www.ncbi.nlm.nih.gov/geo/) with the accession numbers of GSE107475, GSE107311, GSE107156, GSE107125, and GSE115621.

## Supplementary Materials

The following supporting information can be downloaded at: https://www.mdpi.com/article/10.3390/jcdd9090301/s1, Table S1: overlaps of the differentially expressed cell death signature genes; Figure S1: comparisons of cell death processes between iPSC-CM samples; Figure S2: correlation analysis of the cell death processes in the ARVC myocardial samples; Figure S3: standardized ssGSEA enrichment scores of immune cells and pathways; Figure S4: WGCNA of the DEGs between ARVC samples and normal controls; Figure S5: protein-protein interaction (PPI) network of the hub genes of the turquoise module; Supplementary data-sheet, details of analytical results.
