# Supplementary material for: The Landscape of Cell Death Processes with Associated Immunogenic and Fibrogenic Effects in Arrhythmogenic Cardiomyopathy
Source: J Cardiovasc Dev Dis. 2022 Sep 8;9(9):301. doi: 10.3390/jcdd9090301 (PMC9500988; doi:10.3390/jcdd9090301)
Supplement: Supplementary file 1 [file jcdd-09-00301-s001.zip › Supplementary Table and Figures.pdf]

Table S1. Overlaps of the differentially expressed cell death signature genes.

Cell-death category	Overlapped cell-death category	Counts	Gene
Immunogenic cell death	Apoptosis	4	TNF, CASP8， BAX， IL1R1
	Necroptosis	2	TNF, CASP8
	Pyroptosis	2	CASP8, BAX
	Necrosis	3	TNF, CASP8, BAX
	Ferroptosis	0	
	Autophagy	0	
Apoptosis	Necroptosis	3	CASP8, BCL2, TNF
	Pyroptosis	8	TNF, CASP8, BAX, TP53, CYCS, PRKACA, CASP9, CASP3
	Necrosis	5	TNF, CASP8 , BAX, TP53 , BIRC2
	Ferroptosis	1	TP53
	Autophagy	0	
Necroptosis	Pyroptosis	2	TNF, CASP8
	Necrosis	4	TNF, CASP8, SPATA2, CYLD
	Ferroptosis	0	
	Autophagy	0	
Pyroptosis	Necrosis	5	TNF, CASP8, BAX, TP53, NLRP6
	Ferroptosis	1	TP53
	Autophagy	0	
Necrosis	Ferroptosis	1	TP53
	Autophagy	0	
Ferroptosis	Autophagy	0	

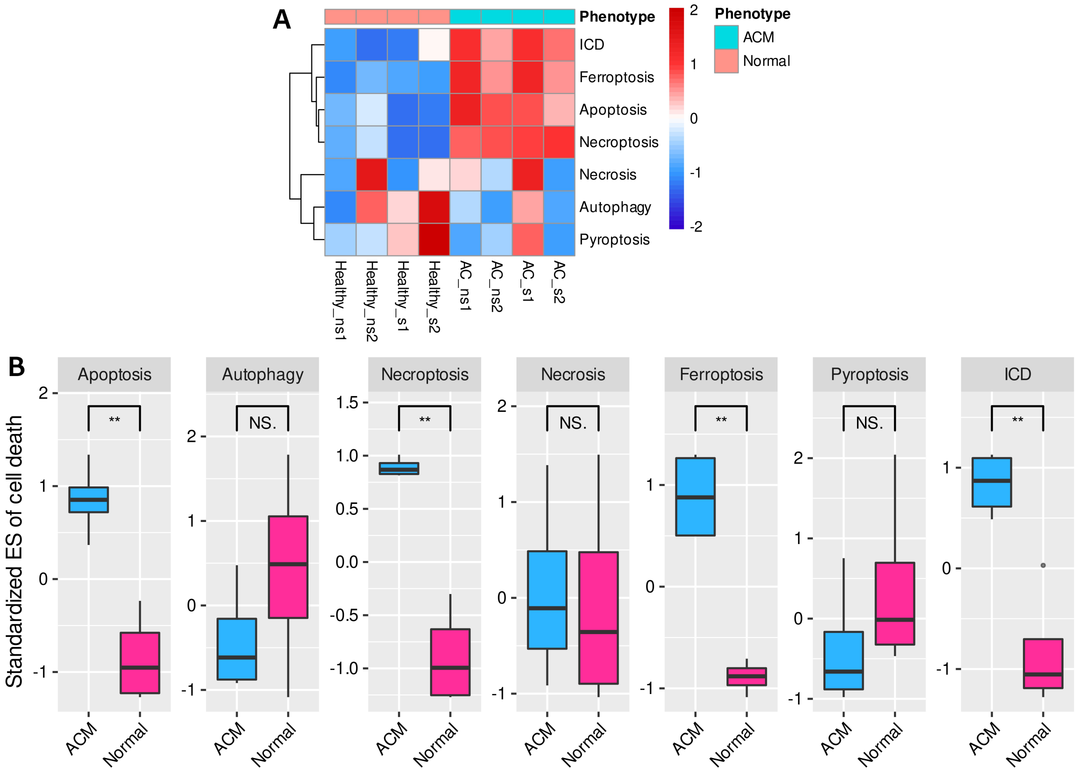

Figure S1. Comparisons of cell death processes between iPSC-CM samples derived from an ACM patient and health control in validation dataset. The levels of apoptosis, necroptosis, ferroptosis, and ICD were significantly higher in ACM iPSC-CM samples than in heath controls, but there was no obvious difference in pyroptosis. ICD, immunogenic cell death. ACM, arrhythmogenic cardiomyopathy; ES, enrichment score of single sample gene-set enrichment analysis.

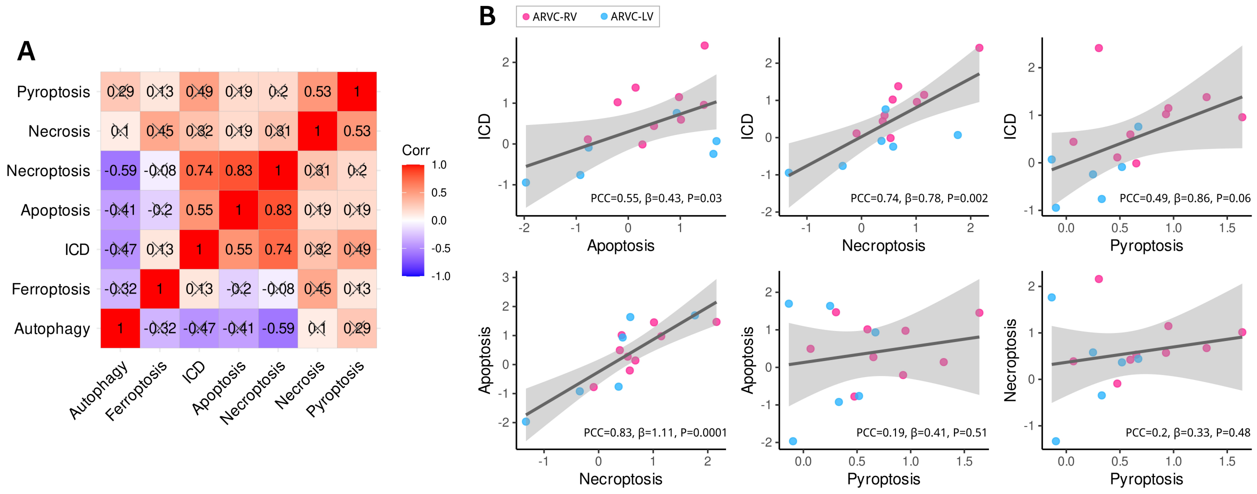

Figure S2. Correlation analysis of the cell death processes in the ARVC myocardial samples. A: Pearson's correlation coefficients (PCCs) matrix with significance test, the symbol "×" means insignificant. B: Linear regression analysis of the up-regulated cell death processes in the ARVC myocardial samples. ICD, immunogenic cell death; ARVC, arrhythmogenic right ventricular cardiomyopathy; ARVC-LV/RV left/right ventricular myocardial samples from ARVC patients.

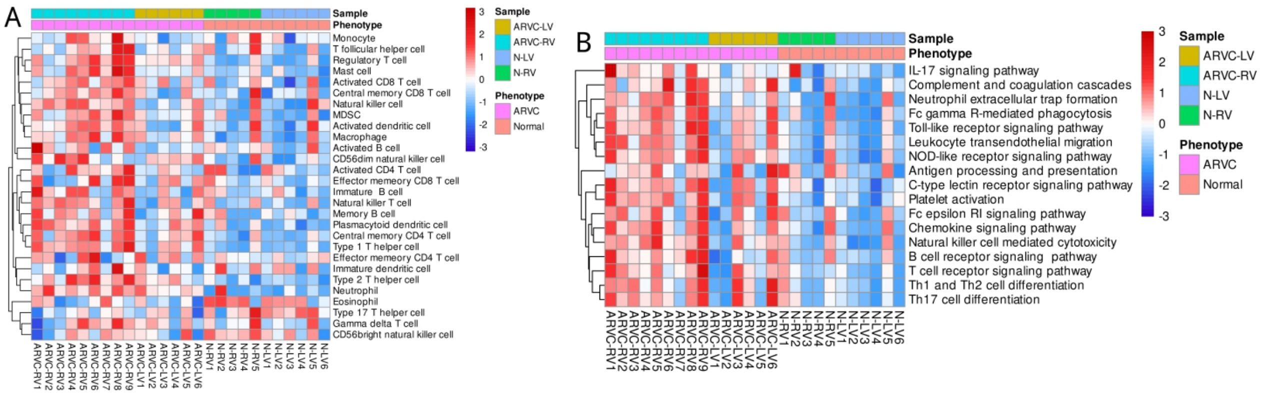

Figure S3. A. Standardized ssGSEA enrichment scores of immune cells for each sample, ARVC samples had generally higher degree of immune cell infiltration than normal controls. B. Standardized ssGSEA enrichment scores of immune pathways for each sample, ARVC samples had generally higher activity of immune pathways than normal controls. ARVC, arrhythmogenic right ventricular cardiomyopathy; ARVC-LV/RV, left/right ventricular myocardial samples from ARVC patients; N-LV/RV, left/right ventricular myocardial samples from health controls; MDSC, myeloid-derived suppressor cell; Th, T helper cell.

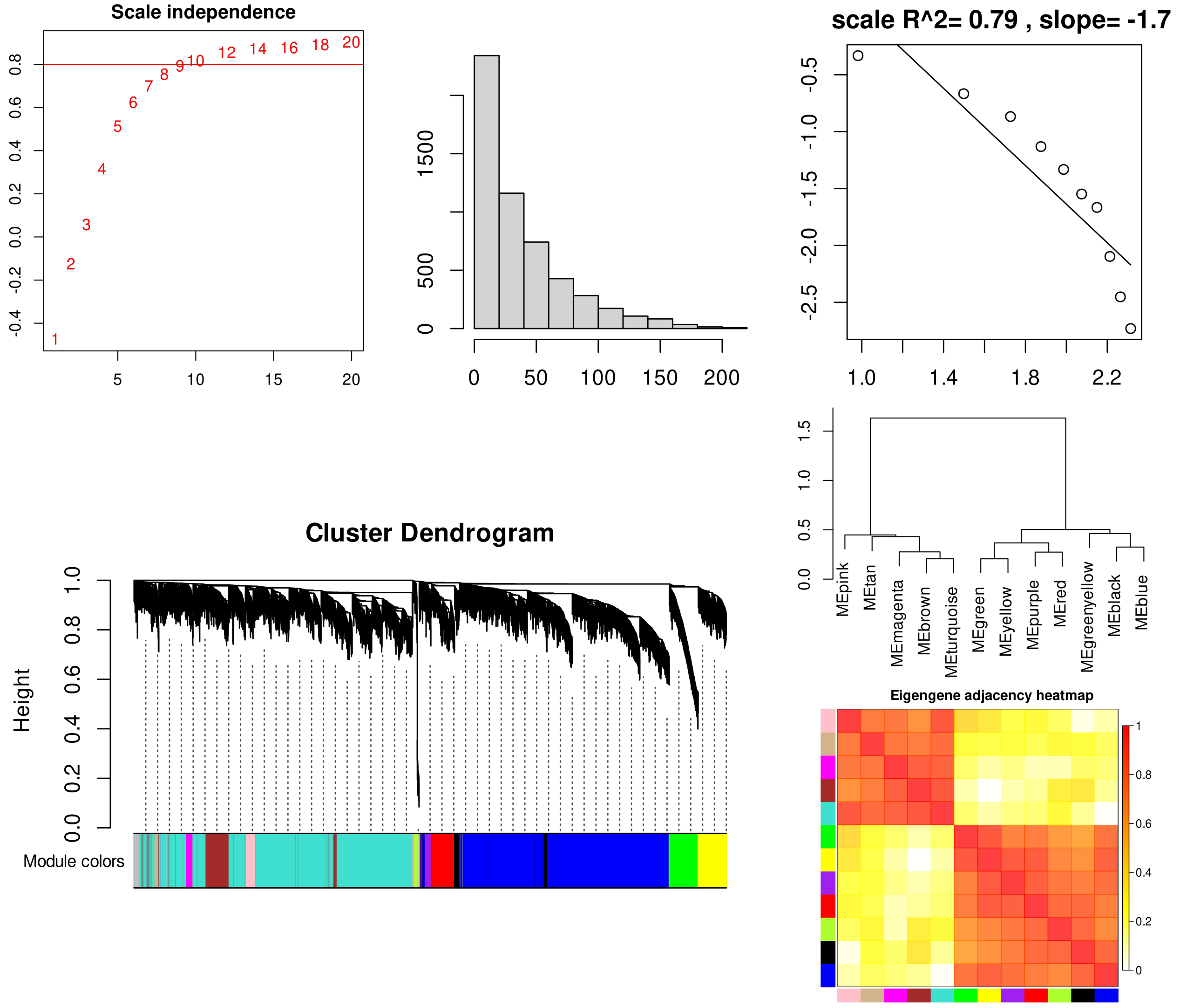

Figure S4. Weighted gene co-expression network analysis of the differentially expressed genes between ARVC samples and normal controls. The optimized soft threshold of 10 was selected to construct a scale-free ($R^2 = 0.79$) co-expression network. Different modules are shown in different colors.

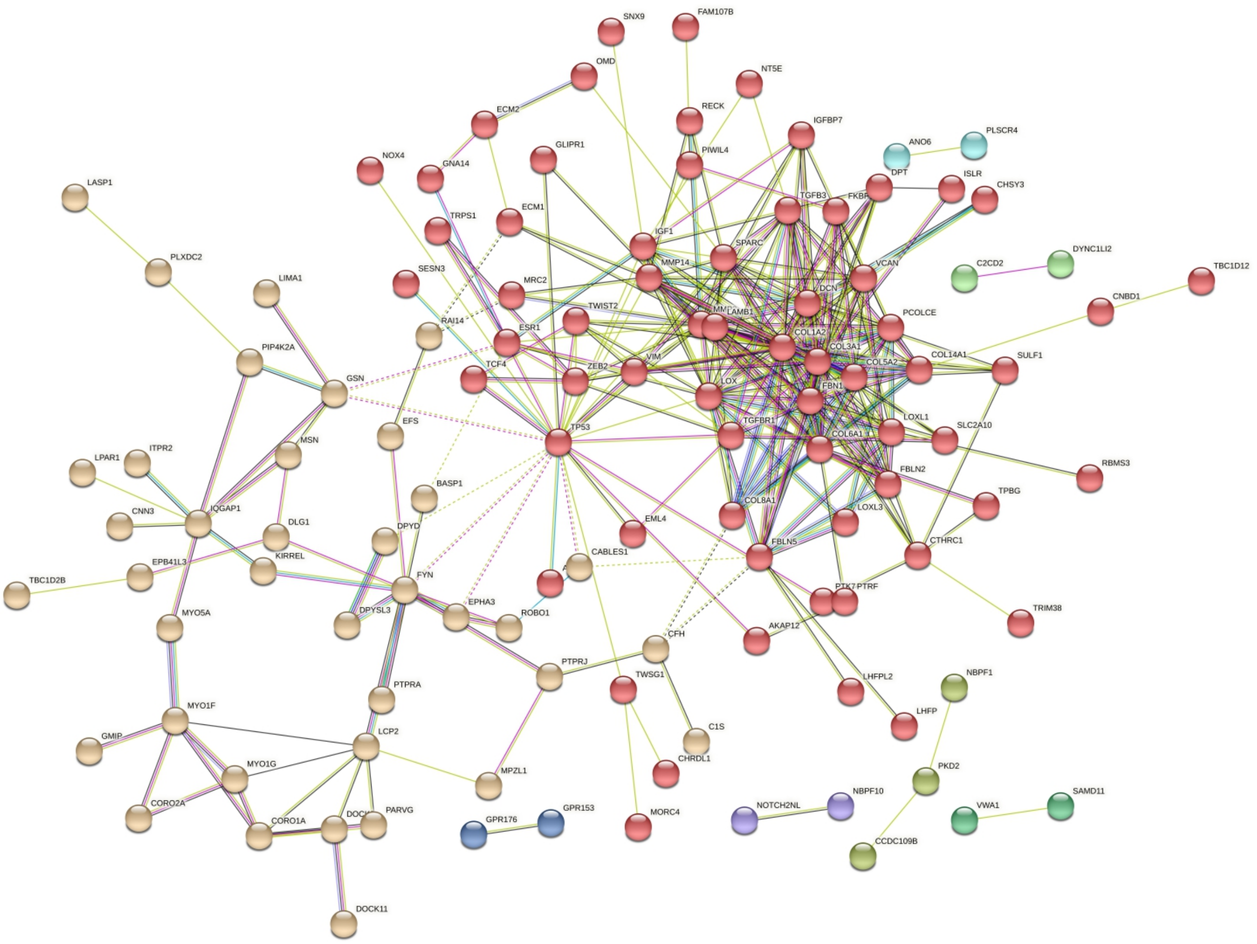

Figure S5. Protein-protein interaction (PPI) network of the hub genes of the turquoise module. The Markov clustering algorithm identified two major PPI groups in the network (colored in red and yellow).
